# Auditory stream segregation using amplitude modulated bandpass noise

**DOI:** 10.3389/fpsyg.2015.01151

**Published:** 2015-08-07

**Authors:** Yingjiu Nie, Peggy B. Nelson

**Affiliations:** ^1^Department of Communication Sciences and Disorders, James Madison UniversityHarrisonburg, VA, USA; ^2^Department of Speech-Language-Hearing Sciences, University of MinnesotaMinneapolis, MN, USA

**Keywords:** amplitude modulation, auditory scene analysis, auditory stream segregation, auditory streaming, bandpass noise, build-up segregation, cochlear implant simulations, sequential grouping

## Abstract

The purpose of this study was to investigate the roles of spectral overlap and amplitude modulation (AM) rate for stream segregation for noise signals, as well as to test the build-up effect based on these two cues. Segregation ability was evaluated using an objective paradigm with listeners' attention focused on stream segregation. Stimulus sequences consisted of two interleaved sets of bandpass noise bursts (A and B bursts). The A and B bursts differed in spectrum, AM-rate, or both. The amount of the difference between the two sets of noise bursts was varied. Long and short sequences were studied to investigate the build-up effect for segregation based on spectral and AM-rate differences. Results showed the following: (1). Stream segregation ability increased with greater spectral separation. (2). Larger AM-rate separations were associated with stronger segregation abilities. (3). Spectral separation was found to elicit the build-up effect for the range of spectral differences assessed in the current study. (4). AM-rate separation interacted with spectral separation suggesting an additive effect of spectral separation and AM-rate separation on segregation build-up. The findings suggest that, when normal-hearing listeners direct their attention towards segregation, they are able to segregate auditory streams based on reduced spectral contrast cues that vary by the amount of spectral overlap. Further, regardless of the spectral separation they are able to use AM-rate difference as a secondary/weaker cue. Based on the spectral differences, listeners can segregate auditory streams better as the listening duration is prolonged—i.e., sparse spectral cues elicit build-up segregation; however, AM-rate differences only appear to elicit build-up when in combination with spectral difference cues.

## Introduction

Auditory stream segregation (also referred to as auditory streaming) occurs naturally in daily life, such as when listening to a talker at a party or when following a melody played by an instrument in an orchestra. Listeners with normal hearing (NH) interpret a mixture of ongoing sounds in such a way that sounds from different sources are allocated to individual sound generators that are perceptually concurrent. Both spectral and temporal differences have been documented as cues that can elicit stream segregation in NH listeners. Studies have employed both pure tones (Bregman and Campbell, 1971; Warren and Obusek, 1972; van Noorden, 1975; Dannenbring and Bregman, 1976a) and bandpass noises (Dannenbring and Bregman, 1976b; Bregman et al., 1999; Nie et al., 2014) to investigate the effect of frequency differences on stream segregation. Bregman et al. (1999) found that interleaved narrowband noises with different amounts of spectral overlap could be perceived as from different auditory streams. Other research has documented that differences in temporal envelopes (Singh and Bregman, 1997; Vliegen et al., 1999; Vliegen and Oxenham, 1999; Grimault et al., 2000, 2001; Roberts et al., 2002) and amplitude modulation rate (Grimault et al., 2002) can induce stream segregation without the presence of spectral cues.

Conflicting findings have been reported on whether cochlear implant users are able to form auditory streams based on auditory signals they perceive with presumably degraded spectral contrasts but well-preserved temporal information. The inconsistency could be attributed to numerous differences among the studies. For example, spectral cue based (Cooper and Roberts, 2009) vs. amplitude modulation based stream segregation (Hong and Turner, 2006) has been evaluated; strength of segregation was measured using self-reported perception (Chatterjee et al., 2006; Marozeau et al., 2013; Böckmann-Barthel et al., 2014) vs. performance-based tasks (Hong and Turner, 2006, 2009; Cooper and Roberts, 2007); tasks with performance promoted by segregation (Hong and Turner, 2009) vs. tasks with performance hindered by segregation (Cooper and Roberts, 2007, 2009, Experiment 1) were used; stimuli involving acoustical signals (e.g., Hong and Turner, 2006) vs. electrical signals (e.g., Chatterjee et al., 2006) were presented to the listeners. Large differences among methodologies make conclusions difficult to interpret.

Even less understood in CI users is one of the key characteristics of stream segregation—the build-up effect which refers to the formation of auditory streams over time following the onset of the mixture of the sound sequences (Bregman, 1990). Chatterjee et al. (2006) and Cooper and Roberts (2009) failed to observe the build-up of streaming in CI users based on the electrode distance equivalent to the spectral differences between stimulus sequences. The conclusion that CI users are unable to segregate auditory streams was drawn by Cooper and Roberts based on the lack of build-up streaming. However, emerging research has suggested the build-up effect may not be observed in NH listeners (Micheyl and Oxenham, 2010b; Deike et al., 2012; Denham et al., 2013). Böckmann-Barthel et al. (2014) further reported comparable course of stream segregation in NH listeners and CI users in that build-up was absent for stimulus tone sequences adequately different in frequency and present when the frequency difference became ambiguous for stream segregation for both groups.

The current study aimed to investigate stream segregation in NH listeners when their listening condition resembled what CI users would commonly experience with degraded auditory cues. Sequences of amplitude modulated bandpass noise used in this study contained two critical cues for CI users—the degraded frequency-difference cue and the supposedly intact AM-rate cue. Unlike previous works (Vliegen and Oxenham, 1999; Hong and Turner, 2009) that varied the amount of inter-stream difference in one cue while controlling for the difference in the other cue, we examined conditions with both inter-stream spectral contrast and amplitude modulation (AM) rate contrast, individually and together. The dual-varying contrasts were studied as a simplistic representation of the co-existing spectral contrast and temporal envelope contrast available to CI users when the stimulus sequences were acoustic pure tones (Böckmann-Barthel et al., 2014).

A performance-based stimulus paradigm (also referred to as an “objective” paradigm) was used to assess stream segregation performance in a listening task. In contrast to a “subjective” paradigm in which stream segregation is assessed based on listeners' report of their perception of one or two streams, an “objective” paradigm is less affected by listener bias, such as listeners having different perceptual criteria for reporting one or two streams. Tasks to identify a violation of temporal regularity have been developed for the performance-based paradigm in different studies (Roberts et al., 2002; Micheyl and Oxenham, 2010a). This study employed a segregation-facilitated paradigm manipulated in such a way that, for better performance, listeners presumably focused attention to segregate auditory streams to identify a temporal violation in the stimulus sequences of noise bursts. The direction of focused attention on segregation, although may not necessarily be (at least completely) controlled by the listener (as suggested by Thompson et al., 2011), is in line with the top-down processing when CI users frequently require mental effort to segregate speech from background noise due to the reduced robustness of auditory cues.

The build-up of stream segregation for bandpass noises, based on spectral and/or AM-rate separations, was also explored in this work. Frequency differences have been confirmed to be a cue for build-up streaming in NH individuals when they listen to pure tone sequences (Anstis and Saida, 1985; Cusack et al., 2004; Thompson et al., 2011). In this study, we investigated whether listeners show build-up of stream segregation when listening to bandpass noises with systematically varied amount of spectral overlap—which reduced the frequency contrast between the potential streams to resemble the spectral interaction of signals delivered via a CI electrode array. It is hypothesized, but not well established, that temporal envelope can also be a cue for segregation build-up. The inconsistent findings on build-up in CI users (as reviewed earlier), in addition to the lack of research on the temporal-envelope based build-up, warrants further research in this area. Understanding how NH listeners use the degraded spectral cues coupled with temporal-envelope cues to form auditory streams and build up auditory stream segregation with attention directed to segregation may help lay basis for further understanding of CI users.

## Experiment 1

### Materials and methods

#### Participants

Ten adult listeners between 19 and 32 years of age, five males, participated in the study. Their hearing thresholds were no greater than 20 dB HL at audiometric frequencies of 250, 500, 1000, 1500, 2000, 3000, 4000, 6000, and 8000 Hz on the right side. The research procedure was approved by the Institutional Review Boards at the University of Minnesota to conduct the experiments on human participants.

#### Apparatus

For all experiments, the stimuli were processed live through a SoundMAX Integrated Digital Audio sound card installed in a Dell Pentium 4 computer. Listeners performed the task in a double-walled sound attenuated booth. Stimuli were generated using a MATLAB script at a sampling rate of 22,050 Hz. The 4th order Butterworth filters were designed and applied to the stimulus via MATLAB.

#### Stimulus sequences

##### Twelve-pair condition (long sequences eliciting build-up)

Twelve repeated pairs of A and B noise bursts were generated as described in our previous work (Nie et al., 2014) with modifications and additional conditions, where A and B bursts were either broadband noise or bandpass noise carrying sinusoidal AM (with 100% modulation depth and fixed phase). They differed either in the center frequency of the noise band, or in the AM-rate, or both.

Each A or B burst was generated with a different sample of noise. The duration of an A or B burst was 80 ms including 8-ms rise/fall ramps. The BRT (i.e., burst repetition time)—defined as an interval between the onsets of two consecutive bursts (i.e., the onsets of an A burst and the B burst proceeding or following the A burst, or the onsets of a B burst and the A burst proceeding or following the B burst)—was 130 ms, while A bursts (excluding the initial one) were jittered from their nominal temporal locations by an amount drawn randomly on each presentation from a rectangular distribution ranging from 0 to 40 ms. The amount of jitter of A bursts was selected based on a pilot study which demonstrated adequate disruption to following the rhythm of A-B pairs. The rationale for presenting B bursts steadily was that B bursts consisted of a passband with a lower frequency range (from 200 to 1426 Hz) which may provide the major information for speech understanding. Bashford and Warren (1987) found NH listeners scored 98% or higher when listening to words and sentences which were lowpass filtered at a cutoff frequency of 1100 Hz. In addition, Whitmal and DeRoy (2012) reported that, for NH listeners, frequencies below 1500 Hz became more important when natural speech was processed through vocoder processing. Therefore, it was of interest to investigate listeners' ability to follow the stream in this lower frequency range considering its importance for speech perception (see the Section on Procedure for details about the task).

**Figure 1:**
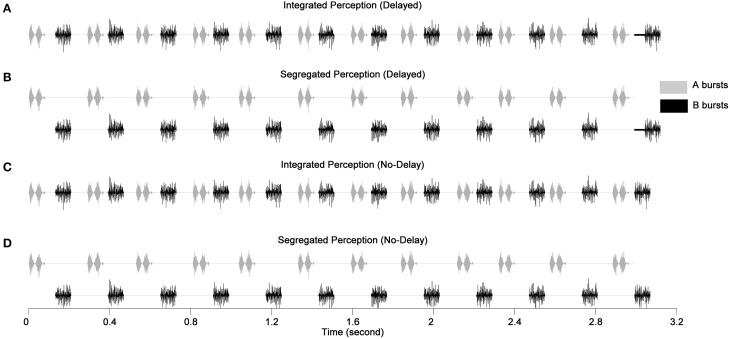
**Illustration of the stimulus paradigm (modified from Nie et al., 2014)**. **(A,B)** Illustrate the delayed sequences with the dark solid lines showing the duration of the delay for the last B burst. **(C,D)** Illustrate the no-delay sequences. **(A,C)** Depict the integrated perception and **(B,D)** depict the segregated perception. The spectral conditions for A and B bursts are A_678_ and B_1234_, respectively. The AM rates shown on the A bursts and B bursts are 25 and 300 Hz, respectively.

Two types of stimulus sequences were adopted differing in the placement of the last B burst as illustrated in Figure 1. In a delayed sequence, the last B burst was delayed from its nominal temporal position by 30 ms, whereas, in a no-delay sequence, the last B burst was advanced by an amount drawn randomly on each presentation from a rectangular distribution ranging from 0 to 10 ms. The total duration was 3.1 s for the delayed sequences and 3.06–3.07 s for the no-delay sequences.

Independent Gaussian noise was generated for the each broadband noise (BBN) burst. To obtain the bandpass noises, the independent Gaussian noise for each noise burst was filtered at cutoff frequencies adopted from the vocoder bands in Fu and Nogaki (2005). Table 1 shows the cutoff frequencies with a resolution of eight bands. The bands were numbered from one to eight corresponding to bands with center frequencies from low to high. The B band was obtained by filtering a Gaussian noise at the low cutoff frequency of band 1 and the high cutoff frequency of band 4; hence the B band (B_1234_) covered the bands 1 through 4 in Fu and Nogaki. With the same method, the higher three bands (e.g., bands 6, 7, and 8) formed another bandpass noise which was presented as one of the A band conditions and coded as Axxx (e.g., A_678_). While the spectrum of the B band was constant (i.e., encompassing the lowest four vocoder bands), the spectra of the A bands covered four conditions, in relationship with the spectrum of B band:

**Table 1:** **Cutoff frequencies of the A and B bands at four spectral conditions and the relationship of the A and B bands with the eight vocoder bands from Fu and Nogaki (2005)**.

Seventy-seven-percent-overlap		**A**_**234**_**: Bands 2, 3, and 4**						
Forty-one-percent-overlap			**A**_**345**_**: Bands 3, 4, and 5**					
Seventeen-percent-overlap				**A**_**456**_**: Bands 4, 5, and 6**				
No-overlap						**A**_**678**_**: Bands 6, 7, and 8**		
	**B bursts: Bands1234**							
**Vocoder band (8-band resolution)**	**1**	**2**	**3**	**4**	**5**	**6**	**7**	**8**
Low cutoff frequency (Hz)	200	359	591	931	1426	2149	3205	4748
	(5.84)	(8.77)	(11.86)	(15.08)	(18.39)	(21.77)	(25.17)	(28.62)
High cutoff frequency (Hz)	359	591	931	1426	2149	3205	4748	7000
								(32.09)

The cutoff boundaries of the vocoder bands in the ERB scale are shown in parentheses.

First, no-overlap—A_678_ B_1234_ (A band consisted of bands 6, 7, and 8 as in Fu and Nogaki).

Second, seventeen percent (17%) overlap—A_456_B_1234_ (A band consisted of bands 4, 5, and 6) with 17.1% overlap in the equivalent rectangular bandwidth (ERB) scale (Glasberg and Moore, 1990), derived from Equation 1.

(1) $$\frac{\begin{array}{l} (\text{high cutoff boundary of B band}\\ \;\text{− low cutoff boundary of A band}) \end{array}}{\begin{array}{l} (\text{high cutoff boundary of A band}\\ \;\text{− low cutoff boundary of B band}) \end{array}}\;\times \text{ 100}\%$$

where the cutoff boundaries were calculated in the ERB scale (Table 1), i.e., $\frac{\left(18.39-15.08\right)}{\left(25.17-5.84\right)}\times 100\%\;=\;17.1\%$.

Third, forty-one percent (41%) overlap—A_345_B_1234_ (A band consisted of bands 3, 4, and 5) with 41.0% overlap in the ERB scale.

Fourth, one hundred percent (100%) overlap—A_BBN_B_BBN_ (both A and B bands consisted of broadband noise).

It should be noted that the slope of the bandpass filters was set at 12 dB/octave to resemble the shallow filter slope in CI users (Anderson et al., 2011). In consequence, the actual band overlap was larger than that calculated using Equation 1.

Four comparisons of AM-rates were applied between A and B bands, as follows. First, unmodulated (AM0-0) with no AM applied to either A or B band; second, no separation in modulation rate (AM25-25) with both A and B bands modulated at a rate of 25 Hz; third, modulation rates 2 octaves apart (AM25-100) with A and B bands modulated at rates of 25 and 100 Hz, respectively; and fourth, modulation rates 3.58 octaves apart (AM25-300) with A and B bands modulated at rates of 25 and 300 Hz, respectively.

##### Three-pair sequences (short sequences providing baseline for evaluating the build-up effect)

Three pairs of A and B bursts (3-pair) were presented for three spectral separations including 100%-overlap (i.e., A_BBN_B_BBN_), 41%-overlap (i.e., A_345_B_1234_), and no-overlap (i.e., A_678_B_1234_). The temporal settings for A and B bursts in a 3-pair sequence were the same as those in a 12-pair sequence with only the first, second, and the last stimulus pairs of a 12-pair sequence preserved.

#### Procedure

In a pilot study, it was observed that the attentional effort required to perform the task was too high for listeners to maintain concentration for a two interval approach due to the length of a stimulus sequence in addition to the substantially reduced cues. Therefore, *d*′ was measured through a single interval yes/no approach. In each interval, either a delayed sequence or a no-delay sequence was presented.

The stimulus sequences were presented monaurally to the right ear through a TDH 49 headphone at 70 dB SPL for each noise burst calibrated based on the root-mean-square value. The task was to determine whether the delayed sequence or the no-delay sequence was presented in each trial. Two response options were given in two graphic boxes on a computer screen, one showing “1 Longer” for the “delayed” option and the other one showing “2 Shorter” for the “no-delay” option. The participants pressed on the keyboard number 1 (for the “delayed” choice) or number 2 (for the “no-delay” choice). Feedback was provided following each response by illuminating the box corresponding to the correct answer on the screen. Participants were allowed to take as much time as they needed to make the selection for each trial.

This task directed listeners to focus attention on segregating two streams in order to reach a better performance. To detect the delayed last B bursts, listeners had to discriminate the prolonged gap between the last two B bursts as opposed to the constant B-to-B gaps of the previous 11 B bursts (See Figure 1 to contrast panels B and D for the difference between the no-delay and delayed sequences). The jittered timing of A bursts introduced uncertainty to an A-to-B gap, thus made an A-to-B gap an ineffective cue for the identification of the delayed B bursts. Hence, listeners had to follow B bursts and ignore A bursts in order to determine the gaps between B bursts. In other words, for better performance, listeners presumably made mental efforts to segregate B bursts from A bursts to form a perceptual stream of B bursts. To sum up, the better a listener could segregate the B stream from A stream, the better he/she could detect the last delayed B burst.

Four blocks of 70 trials were run for each condition with a 50% chance of occurrence for either the signal sequence (i.e., delayed sequence) or the reference sequence (i.e., no-delay sequence). The first 10 trials were designed to facilitate the listeners forming and maintaining stream segregation. From the last 60 trials, the hit rate and false alarm rate were calculated, which were used to compute a *d*′. Ceiling performance (i.e., 100% for hit rate and 0% for false alarm rate) was reached in 7% of the total number of blocks in all listeners and was corrected using Equations 2 and 3 (Macmillan and Creelman, 2005).

(2) $$Hit\;Rate=1-\frac{1}{\text{2S}}\times \text{100}\%$$

(3) $$False\;Alarm\;Rate=\frac{1}{\text{2N}}\times \text{100}\%$$

where S and N represent the total possible numbers of trials presented for signal and reference sequences, respectively.

Following an initial training session (see Familiarization for detail), participants were presented stimulus sequences in a random order of the spectral separation/duration condition. Four AM-rate separations were randomly nested under each spectral/duration condition. Participants completed their sessions across multiple days, one or two 1.5-h sessions each day. They were encouraged to take a 5-min break after 2 or 3 blocks. Due to time constraints, six participants participated in the 100%-overlap/3-pair conditions; among these six participants, four participated in the no-overlap/3-pair conditions. All 10 participants participated in the rest of the conditions.

#### Familiarization

##### Training session

The first 1.5-h session was designed for training purposes. The structure of the stimulus sequences was described to the participants verbally and with a schematic illustration. They were encouraged to follow the subsequence consisting of elements that were presented steadily. Only 12-pair sequences were used in this session.

Participants were initially presented with the presumably easiest condition—no-overlap. All participants reported perceiving segregated streams in this block. Additional blocks of the same condition were undertaken until a participant's *d*′ was larger than 2. Then, the spectral separation was decreased progressively with the AM-rate separation of either AM25-300 or AM0-0 applied to each of the spectral conditions. With 30–45 min of familiarization (1–5 blocks for each of the spectral conditions), all participants reported consistent segregation perception throughout at least one block in each of the spectral conditions of no-overlap, 17%-overlap, and 41%-overlap. However, they reported difficulties in holding the segregated perception for the 100%-overlap condition with AM25-300 separation, for which participants needed 45–60 min to repeat 8–12 blocks.

##### Experimental sessions

Prior to data collection in each experimental session, participants practiced the task with two 40-trial blocks of 12-pair sequences, one for the no-overlap condition and one for the 100%-overlap condition with the AM separation of AM25-300. All participants reported the capability of holding segregated perception throughout the block of no-overlap condition. More blocks were presented if participants reported absolutely no perception of segregation for the 100%-overlap condition until they reported intermittent segregation perception.

### Data analysis

IBM SPSS statistics version 21 was used for data analysis and means and standard errors are reported in the results. Data were analyzed using the linear mixed-model approach which is specified in the Results Section for readability.

### Results and discussion

#### Auditory stream segregation based on spectral separation and AM-rate separation

Listener performance measured with 12-pair stimulus sequences was analyzed via a linear mixed-model. The spectral separation and AM-rate separation were assessed for the fixed repeated effect, while the subject variables in the model included participants and the repetitions of *d*′ measures within each observational unit (i.e., a given AM-rate separation nested in a spectral separation).

**Figure 2:**
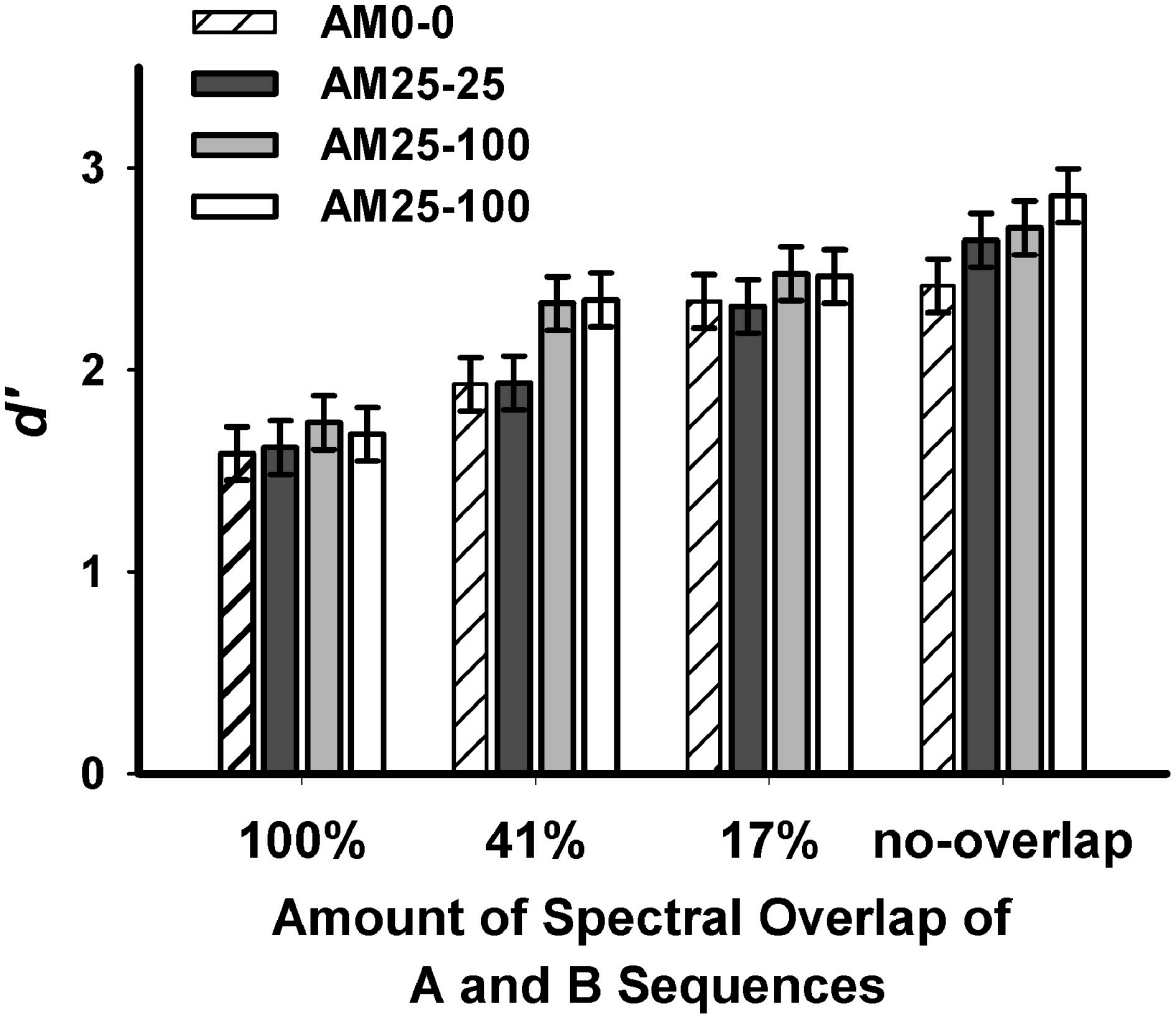
**Mean *d*′-values in various spectral and AM-rate conditions of the stimuli sequences collapsed across the 12-pair sequence duration in Experiment 1 (error bars represent ±1 standard errors around the means)**.

Figure 2 shows mean *d*′-values for the 12-pair sequences under each spectral/AM-rate separation. Significant differences were found for spectral separation [*F*_(3, 585)_ = 77.09, *p* < 0.0001], and AM-rate separation [*F*_(3, 585)_ = 7.61, *p* < 0.0001]. No significant interaction was seen between spectral separation and AM-rate separation [*F*_(9, 585)_ = 1.01, *p* = 0.4317]. These findings suggest that when either the spectral separation or the AM-rate separation increases, listeners can better segregate ongoing interleaved stimuli into different perceptual streams.

##### Pairwise comparisons between spectral separations

Pairwise comparisons with Bonferroni adjustment showed progressively increased *d*′-values (Table 2) as spectral separation between A and B subsequences increased from 100%-overlap to no-overlap (p < 0.001 for each comparison).

**Table 2:** **Mean *d*′-values in the four spectral conditions for pooled data across AM-rate conditions and mean *d*′-values in the four AM-rate conditions for pooled data across spectral conditions**.

**SPECTRAL CONDITION**				
	**100% overlap**	**41% overlap**	**17% overlap**	**No-overlap**
*d*′	1.66 (0.07)	2.13 (0.08)	2.40 (0.06)	2.67 (0.06)
**AM-RATE CONDITION**				
	**AM0-0**	**AM25-25**	**AM25-100**	**AM25-300**
*d*′	2.07 (0.07)	2.13 (0.07)	2.31 (0.08)	2.34 (0.07)

The measured standard errors are shown in parentheses.

##### Pairwise comparisons between AM-rate separations.

The mean *d*′−values for the four AM-rate conditions are shown in Table 2. With Bonferroni adjustment, better performance was revealed for AM25-300 than for AM25-25 (*p* = 0.0134) and AM0-0 (*p* = 0.0006). Performance for AM25-100 was also significantly better than for AM25-25 (*p* = 0.0446) and for AM0-0 (*p* = 0.0025). No difference was shown between the AM0-0 and AM25-25 conditions or between the AM25-100 and AM25-300 conditions (*p*>0.9999 for either comparison). These results suggest that when the AM-rate difference is 2 octaves or larger, it can be a cue for listeners to segregate the interleaved A and B noise bursts into two auditory streams.

#### Build-up effect: stream segregation based on 3- vs. 12-pair stimulus sequences

Comparison of results for 3- and 12-pair stimuli revealed the extent of segregation build-up. For a given participant, a spectral separation (including the four AM-rate separations nested under it) for the 12-pair stimulus sequences was excluded from the mixed model of analysis, if it was not tested for the 3-pair stimulus sequences. Repeated factors were spectral separation and AM-rate separation, with subject variables of participants, duration of sequences, and repetitions of the *d*′ measure within a given observational unit. Three independent factors were assessed including sequence duration (12-pair vs. 3-pair), spectral separation (no-overlap, 41%-overlap, and 100%-overlap), and AM-rate separation (AM25-300, AM25-100, and AM25-25).

**Figure 3:**
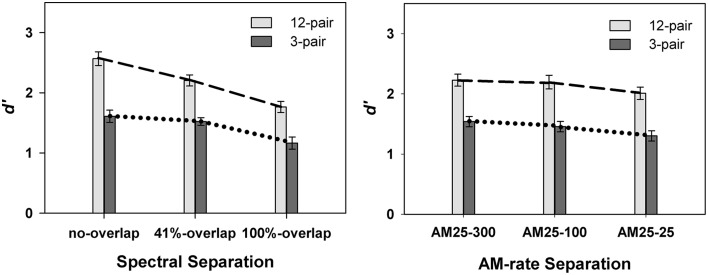
**Contrasts of mean *d*′-values in Experiment 1 between 12-pair and 3-pair stimulus sequences for the three spectral separations (left panel) and for the three AM-rate separations (right panel) (error bars represent ± one standard errors)**. Significance was found for the interaction of spectral separation X sequence duration, but not for the interaction of AM-rate separation X sequence duration.

**Figure 4:**
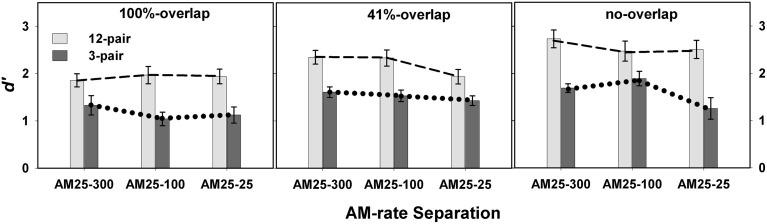
**Mean *d*′-values for 12-pair and 3-pair conditions in Experiment 1 are illustrated as a function of AM-rate separation in three spectral separation conditions: no-overlap, 41%-overlap, and 100%-overlap**. Error bars represent ± one standard errors.

Overall, listeners showed better performance in the 12-pair conditions (mean = 2.25 ± 0.10) than in the 3-pair conditions (mean = 1.48 ± 0.10) [*F*_(1, 86)_ = 27.80, *p* < 0.0001]. A significant interaction was revealed for spectral separation X duration [*F*_(2, 427)_ = 5.13, *p* = 0.0063] (left panel in Figure 3), but not for AM-rate separation X duration [*F*_(2, 398)_ = 0.34, *p* = 0.7137] (right panel in Figure 3). However, the three way interaction of spectral separation X AM-rate separation X duration was found to be significant [*F*_(12, 399)_ = 2.04, *p* = 0.0407] (Figure 4).

These results indicate that, listeners were able to segregate the A and B streams better for the 12-pair sequences. In addition, the significant interaction of spectral separation and sequence duration revealed a steeper slope for 12-pair sequences in the performance / spectral separation function. This suggests greater build-up effect of stream segregation for a larger spectral difference. In other words, spectral separation elicited the build-up effect and facilitated stream segregation.

While the overall non-significant interaction of AM-rate separation and sequence duration indicates limited to no build-up of stream segregation as the AM-rate separation increased, the significant three-way interaction (spectral separation X AM-rate separation X sequence duration) suggests the effect of AM-rate on build-up may be spectral-separation dependent. Figure 4 reveals greater increase in *d*′ scores with the increase of AM-rate-separation for the 12-pair stimulus sequences than for the 3-pair sequences when the two stimulus subsequences were spectrally different (i.e., in the 41%-overlap or no-overlap). In addition, this trend appears more salient in the 41%-overlap than in the no-overlap, suggesting a possible (if not all, stronger) interaction of AM-rate-separation and sequence duration in the 41%-overlap—the condition with less inter-subsequence spectral separation.

## Experiment 2

The objective of this experiment was to confirm the AM-rate cue for build-up as suggested by the interaction of spectral separation and AM-rate separation for the build-up segregation in Experiment 1. We assessed listeners' performance when the two stimulus subsequences were more spectrally overlapping than what had been tested in Experiment 1. The Apparatus in this experiment was identical to that in Experiment 1.

### Materials and methods

#### Participants

Five female listeners between 19 and 44 years of age who did not participated in Experiment 1 participated in this experiment. Their hearing thresholds met the criteria in an audiometric test as stated in Experiment 1.

#### Stimulus sequences and procedure

The stimulus sequences were generated using the same approach as in Experiment 1, with B bursts presented at equal intervals while A bursts pseudo-randomly presented between the two B bursts. Stimulus sequences of 12- and 3-pairs of A and B bursts were used to examine the build-up effect. Only two spectral separations between the A and B subsequences were studied; the first was a 77%-overlap condition, with the cutoff frequencies corresponding to Bands #2–4 in Fu and Nogaki (2005) as shown in Table 1, which was not tested in Experiment 1. The second was a 41%-overlap condition, which was repeated from Experiment 1. The selection of these two spectral separations was to confirm the hypothesis suggested in Experiment 1 that AM-rate cues for build-up may be more salient when spectral cues were moderate. Thus, a total of four duration/spectral conditions were assessed in this experiment in a random order for each participant. All four AM-rate separations (AM25-300, AM25-100, AM25-25, and AM0-0) were nested under each of these four conditions.

The participants followed the Familiarization and general procedures adopted from Experiment 1^1^, but attended fewer experimental sessions due to the reduced number of conditions.

### Data analysis

IBM SPSS statistics version 21 was used for data analysis. The same mixed linear analysis model from Section Build-up effect: Stream Segregation Based on 3- vs 12-Pair Stimulus Sequences in Experiment 1 was applied to assess three independent factors—sequence duration (12-pair and 3-pair), spectral separation (41%-overlap and 77%-overlap), and AM-rate separation (AM25-300, AM25-100, and AM25-25).

### Results and discussion: build-up stream segregation based on AM-rate separation on stimulus sequences with minimal to moderate spectral separations

**Figure 5:**
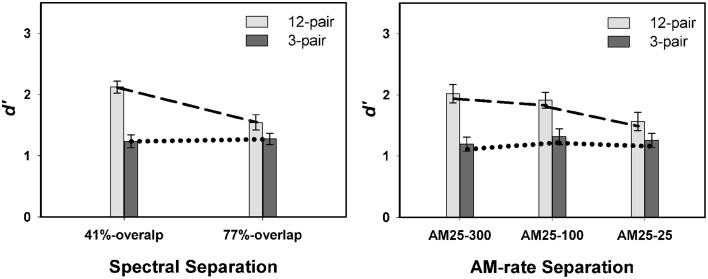
**Contrasts of mean *d*′-values between 12-pair and 3-pair stimulus sequences in Experiment 2 for the two spectral separations (left panel) and the three AM-rate separations (right panel) (error bars represent ± one standard errors)**. Significance was found for the interactions of spectral separation X sequence duration and AM-rate separation X sequence duration.

Listeners showed better performance in the 12-pair conditions (mean = 1.83 ± 0.12) than in the 3-pair conditions (mean = 1.26 ± 0.12) [*F*_(1, 40)_ = 10.97, *p* = 0.0020]. Interactions of sequence duration were found to be significant both with spectral separation [*F*_(1, 200)_ = 14.29, *p* = 0.0002] (left panel in Figure 5) and with AM-rate separation [*F*_(2, 200)_ = 3.33, *p* = 0.0377] (right panel in Figure 5). However, the three way interaction of spectral separation X AM-rate separation X duration was not significant [*F*_(4, 200)_ = 0.57, *p* = 0.6834]. The results exhibited greater *d*′ increase from 3-pair to 12-pair stimulus sequences as either inter-subsequence spectral separation or AM-rate separation increased, consistent with the notion that spectral and AM-rate contributed to the build-up stream segregation when the spectra of the stimulus subsequences were minimally to moderately separate. The non-significant three-way interaction (sequence duration X spectral separation X AM-rate separation) indicates AM-rate contributed to the build-up effect to a comparable level between the two spectral separations in this experiment.

## Discussion

In the current study, we used an objective stimulus paradigm to show that NH listeners can voluntarily segregate two interleaved subsequences of noise bursts into two auditory streams based on inter-subsequence differences in spectrum, or in AM-rate, or in both, when the subsequences are presented at different rhythms. This result is consistent with previous findings that both spectral and temporal cues can elicit stream segregation. Our results extend previous findings to further describe the build-up of segregation based on spectral separation or AM-rate separation.

### Stream segregation of wide-bandpass noises based on spectral separation and AM-rate separation

Consistent with Bregman et al. (1999) who found that interleaved narrow band noises with different center frequencies could elicit stream segregation based on a subjective paradigm, our findings showed that spectral separation for wide-bandpass noises could induce stream segregation with an objective segregation-directed paradigm. It may be noted that for stimuli in Bregman et al's study, an intensity difference between the two potential streams occurred due to the study design to maintain equal pitch strength for the two streams. The intensity difference may have been used as a cue for segregation (van Noorden, 1975; Hartmann and Johnson, 1991). The current study equated the intensity for both sets of noise bands and confirmed that the bandpass noise, with a sufficient spectral separation, can be segregated into different streams. In addition, the present study also showed the expected weaker strength of segregation associated with more spectral overlap, which was in contrast with the trend revealed in Figure 2 in Bregman et al. This discrepancy may be due to the different methods used to vary the amount of spectral overlap: The amount of spectral overlap covaried with the center frequency difference between the A and B bandpass noises in the current study; whereas, the center frequency distance was fixed while the amount of spectral overlap varied in Bregman et al.

AM-rate differences were also found to aid stream segregation in the current study. This finding is consistent with the findings of Grimault et al. (2002) who reported that AM-rate differences can elicit stream segregation for the AM rates of 100 Hz and higher. In addition, the present study extends findings to lower AM-rates more relevant for speech-like stimuli, and describes the role of AM-rate difference in segregating streams of bandpass noise. Previous reports in the literature only used broadband noise carriers (Grimault et al., 2002; Hong and Turner, 2009).

An interesting contrast between spectral and AM-rate cues for stream segregation was revealed: The effect of AM-rate difference on stream segregation appeared to approach maximal strength at the 2-octave separation, which was demonstrated by the comparable performance for the AM25-100 (i.e., 2-octave separation) and AM25-300 (i.e., 3.58-ocatve separation) conditions. Similar amounts of AM-rate separation for the “knee-point” that elicits maximal strength for the segregated percept have also been observed in Grimault et al. (cf, Figure 1, 2002). Conversely, increased spectral separations progressively elicited stronger percept of stream segregation. From such a contrast, we infer that, changes in spectral separation may be more perceptually salient for stream segregation than changes in AM-rate separation. As our study did not incorporate experimental manipulations to assess the relative salience of the spectral and AM-rate cues for segregation, future studies would be necessary to explore how stream segregation is affected differently by the relative perceptual salience of one cue vs. the other.

### Build-up stream segregation of wide-bandpass noises based on spectral separation and AM-rate separation

In the current study, adequate spectral separation and AM-rate difference were found to elicit build-up of stream segregation with the present objective paradigm that directs listeners' attention to stream segregation. This strengthens the conclusion that listeners are able to use these cues for segregation. With the experimental design, the duration of a 3-pair sequence was 0.73–0.77 s, which was presumably not long enough to generate perceptual segregation, whereas, a 12-pair sequence with a duration of over 3 s was presumably able to induce better performance than a 3-pair sequence would, if listeners experienced stream segregation. The results evaluating the build-up effect in both experiments showed higher *d*′-values for 12-pair sequences than for 3-pair sequences, confirming that stream segregation was elicited by the AM bandpass noise (Possible confounding factors that may also induce a higher *d*′ for the 12-pair sequences will be discussed later).

Consistent with the Thompson et al. study (2011), build-up of stream segregation was found to be facilitated by spectral separations in the current study when listeners attended to segregating different streams and the effect could be extended to bandpass noise stimuli. The inter-subsequence spectral separations selected in the current study continued to elicit the build-up effect, up to the no-overlap spectral condition. It should be noted that, this finding is inconsistent with earlier studies showing absent build-up stream segregation based on electrode separations (equivalent to spectral differences) in most CI users (Chatterjee et al., 2006; Cooper and Roberts, 2009). The discrepancy may be partially attributed to the use of different paradigms in these studies: A segregation-promoted objective paradigm was adopted in the current study, in contrast to a subjective paradigm in the Chatterjee et al study and an integration-promoted objective paradigm in the Cooper and Roberts report.

Furthermore, our study revealed a spectral separation dependent effect for build-up of stream segregation based on AM-rate differences. That is, in combination with minimal-to-moderate spectral differences of the two sets of bandpass noise, the AM-rate separation elicited the build-up effect. Although the AM-rate cue for build-up diminished when all the spectral conditions were pooled in the analysis [including identical spectrum (100%-overlap), moderate (41%-overlap), and large spectral separations (no-overlap)], the spectral separation dependency was still noted. This finding suggests that listeners may somehow incorporate both cues together when one alone may be ambiguous, showing an additive effect of using spectral and AM-rate cues to improve stream segregation over time. The additive effect in auditory stream segregation has been reported for stable temporal patterns in the stimulus sequences and the inter-stream physical property differences (Denham et al., 2013). In this study, we observed an additive effect on build-up stream segregation from two inter-stream physical property differences.

### Possible alternative explanations of the results

It might be argued that these results could be explained by stream segregation based on other cues or mechanisms not involving stream segregation. Three alternative explanations are considered as follows. First, rhythmic cues in the stimulus sequences facilitated stream segregation. The listeners were found to perform better with the 12-pair vs. the 3-pair sequences when A and B bursts were identical (i.e., in the conditions of 100%-overlap/AM0-0 and 100%-overlap/AM25-25) [*F*_(1, 5)_ = 8.66, *p* = 0.03218, using repeated measure ANOVA]. This can be explained by rhythm-based stream segregation, in that attention to the rhythmic regularity was used by the listeners to segregate the steadily-presented B stream from the irregular A stream. This can occur even in the absence of any other cues when the global coherence of the sequence is low due to the use of fresh noise for every burst (Agus and Pressnitzer, 2013). The rhythmic cue has been reported to enable voluntary stream segregation (for a review, see Bendixen, 2014) in both behavioral (Devergie et al., 2010) and neurophysiological (Nie et al., 2014) studies. However, such rhythm-based segregation cannot explain the observation that the *d*′-values were greater for larger spectral and AM-rate separations. This improved performance confirmed that listeners segregated the A and B streams based on spectral and AM-rate differences. Hence, the mean *d*′-value of 1.5 in conditions of 100%-overlap/AM0-0 and 100%-overlap/AM25-25 reflects a baseline performance for the 12-pair condition involving rhythm-based stream segregation. A *d*′ greater than 1.5 reflects the additional effect resulting from stream segregation based on spectral or AM-rate cue measured with the current paradigm.

Second, it is possible that listeners were able to detect the signal sequence by focusing on the last pair of A and B bursts (instead of focusing on the ongoing sequence). To examine this hypothesis, an ideal observer was simulated to detect the delayed B burst with stimuli only consisting of the last pair of A and B bursts (See Appendix in Supplementary Materials for details). The behavioral performance at two AM-rate separations (AM0-0 and AM25-25) in the 100%-overlap spectral condition for the 3-pair sequences (with respective mean *d*′−values of 1.09 and 1.07) was found comparable to that of an ideal observer whose *d*′ ranged between 0.74 and 1.40. With the identical A and B bursts, the listeners must perform the task by discriminating the A-B/B-A gaps; thus no stream segregation was involved. The comparable performance between an ideal observer and behavioral data suggests limited or no reliance on simple gap detection for the last pair of A-B bursts, and supports the stream segregation hypothesis.

Third, the AM could have introduced spectral cues by generating distortion products. It is presumed that if the additional spectral components had been cues, faster modulation rates (100 and 300 Hz) applied to the B bursts would have generated spectral components spread out over more frequencies. The power spectrum of a B burst was calculated for all conditions. The power differences between the modulated and unmodulated bursts were within 1 dB in the region below 1 kHz, which suggested very limited perceivable differences. However, a recent study (David et al., 2014) has shown that very small spectral cues (a few dB difference in excitation pattern) could elicit obligatory streaming. It is then difficult to completely rule out that a 1 dB difference could not elicit voluntary streaming based on the current data set.

### Implications for cochlear implant users

The current study demonstrates that NH listeners are able to segregate amplitude modulated wide-bandpass noises with impoverished spectral difference cues into two auditory streams when they focus attention on segregation. It is further found that NH listeners are able to build up stronger stream segregation based on AM-rate differences in addition to the spectral differences. The results suggest that CI users might segregate different auditory streams if the spectral and modulation rate differences alone are adequately large. It further suggests that the build-up effect may be seen in CI users using spectral and AM-rate cues interactively when the task directs attention focused on stream segregation.

### Conflict of interest statement

The authors declare that the research was conducted in the absence of any commercial or financial relationships that could be construed as a potential conflict of interest.

## Footnotes

^1^A total 60 trials with the *d*′ computed from the last 50 trials were presented to the subjects in Experiment 2 for each of the four blocks of stimuli in a given condition.
