# Supplementary material for: Auditory stream segregation using amplitude modulated bandpass noise
Source: Front Psychol. 2015 Aug 7;6:1151. doi: 10.3389/fpsyg.2015.01151 (PMC4528102; doi:10.3389/fpsyg.2015.01151)
Supplement: Supplementary file 1 [file DataSheet1.DOCX]

APPEDIX: CALCULATION OF AN IDEAL OBSERVER’S $d'$ BASED ON GAP DISCRIMINATION

The signal distribution (S) with the delayed B bursts as in the delayed sequences and the noise distribution (N) with jittered-advanced B bursts as in the no-delay sequences were created. N is a rectangular distribution ranging from 0 to 90 ms in the silent gap between the offset of the A burst and the onset of the B burst with a mean of 45 ms. This range was derived in the following way: In the noise (reference) pairs of A and B bursts, the normal gap between A and B bursts is 50 ms; the jitter of ±40 ms on A bursts results in a range of 50±40 ms—ranging from 10 to 90 ms—in A-B gaps; the random advancing of B bursts in a range from 0 to 10 ms made the effective A-B gaps ranging from 0 to 90 ms with a mean of 45 ms. S is a rectangular distribution with ranging from 40 to 120 ms with a mean of 80 ms. This is the range of A-B gaps which was derived from adding the 30-ms delay of B bursts on the A-B gaps ranging from 10 to 90 ms resulted from the jitter of ±40 ms on A bursts. The possibility of a value ranging between 40 and 120 ms is from the S distribution was estimated with 500,000 trials; another independent 500,000 trials were analyzed to estimate the possibility of a value ranging between 0 and 90 ms is from the N distribution. The summation of the two probabilities renders the percent correct out of one million trials. The $d'$ for an ideal observer was derived from the percent correct and was approximately 1.07. The standard error of the $d'$ from the simulation was estimated based on 240 observational trials (120 trials each for signal sequences and non-signal sequences) in the behavioral experiment using the equations proposed by Gourevitch and Galanter ([1967](#_ENREF_22)) (Equation 4).

$var\left( d^{'} \right)=\frac{H(1-H)}{Ns{[\phi\left( H \right)]}^{2}}+\frac{F(1-F)}{Nn{[\phi\left( F \right)]}^{2}}$ (4)

where var stands for variance, H for hit rate, F for false alarm rate, Nn for the number of trials with non-signal sequences, Ns for the number of trials with signal sequences, ϕ(H) and ϕ(F) for the heights of the normal density functions at the Z scores of H and F. The function ϕ can be calculated by Equation 5.

$\emptyset\left( p \right)=\frac{1}{\sqrt{2\pi}}e^{-\frac{1}{2}{z(p)}^{2}}$(5)

where z(p) is the Z score of a probability.

The estimated standard error (σ) is the square root of the variance, which was approximately 0.1705. The 95% confidence interval with the center $d'$ at 1.07 is 1.07±1.96 σ, that is, 1.07±0.33, and the resultant range is 0.74~1.40.
